# Predictors for fatal human infections with avian H7N9 influenza, evidence from four epidemic waves in Jiangsu Province, Eastern China, 2013‐2016

**DOI:** 10.1111/irv.12461

**Published:** 2017-07-26

**Authors:** Wang Ma, Haodi Huang, Jian Chen, Ke Xu, Qigang Dai, Huiyan Yu, Fei Deng, Xian Qi, Shenjiao Wang, Jie Hong, Changjun Bao, Xiang Huo, Minghao Zhou

**Affiliations:** ^1^ School of Public Health Nanjing Medical University Nanjing China; ^2^ Jiangsu Provincial Center for Disease Control and Prevention Nanjing China; ^3^ School of Public Health Wannan Medical College Wannan China

**Keywords:** Avian influenza, fatal infection, H7N9, predictor, risk factor

## Abstract

**Background:**

Four epidemic waves of human infection with H7N9 have been recorded in China up to 1 June 2016, including in Jiangsu Province. However, few studies have investigated the differences in patients' characteristics among the four epidemic waves, and the analyses of factors associated with fatal infection lacked statistical power in previous studies due to limited sample size.

**Methods:**

All laboratory‐confirmed A(H7N9) patients in Jiangsu province were analysed. Patients' characteristics were compared across four waves and between survivors and those who died. Multivariate analyses were used to identify independent predictors of death.

**Results:**

Significant differences were found in the lengths of several time intervals (from onset of disease to laboratory confirmation, to onset of ARDS and respiratory failure, and to death) and in the development of heart failure. The proportions of overweight patients and rural patients increased significantly across the four waves. Administration of glucocorticoids and double‐dose neuraminidase inhibitors became the norm. Predictors of death included complications such as ARDS, heart failure and septic shock, administration of glucocorticoids, and disease duration.

**Conclusion:**

Characteristics of H7N9 patients and clinical treatment options changed over time. Particular complications and the use of particular treatment, along with disease duration, could help clinicians predict the outcome of H7N9 infections.

## 1 INTRODUCTION

Since 2013 when the A(H7N9) virus emerged,^1^ a total of 793 laboratory‐confirmed human cases have been reported globally up to 19 July 2016. There have been 319 reported deaths, a fatality rate of 40%.^2^ The novel virus was initially identified in the Yangtze River delta situated in Eastern China and a majority of cases were reported from this district,^3^ which comprises Jiangsu, Zhejiang and Shanghai Provinces/Municipalities. The virus has raised severe concerns due to its ability of binding to receptors in the upper respiratory tract^4^ and the high death rate in human infections. Several published studies have presented preliminary analyses of the risk factors or predictors for fatal human infections with H7N9.^5^, ^6^, ^7^ However, statistical analyses, especially multivariate analyses, were underpowered in most studies due to small sample size (≤40 cases) or patients' limited epidemiological/clinical information. As of 1 June 2016, the H7N9 virus has caused four epidemic waves in Jiangsu Province, resulting in a total of 103 laboratory‐confirmed H7N9 cases with 47 deaths. This study aims to comprehensively identify the epidemiological or clinical predictors of death in human infections with H7N9 using multivariate analyses, based on a relatively large sample size obtained from Jiangsu province from 2013 to 2016.

## 2 METHODS

### 2.1 Subjects

All laboratory‐confirmed human infections with H7N9 are reported through a national system for reporting of notifiable infectious diseases.^8^ Demographic, epidemiological and clinical information on patients infected with H7N9 was collected using standardized questionnaires by local CDC staff or trained clinical doctors in Jiangsu Province and was reported through this system. All patients infected with H7N9 as of 1 June 2016 in Jiangsu Province were included in this analysis.

### 2.2 Ethic statement

The National Health and Family Planning Commission ruled that the collection of data from cases of H7N9 was part of the public health investigation of an emerging outbreak, and thus, the investigation was exempt from institutional review board assessment.^8^ Patients' information was collected and reported to Jiangsu Provincial CDC and China CDC through a national system for reporting of notifiable infectious diseases. Jiangsu Provincial CDC is responsible for checking and monitoring the reported information and will take part in patients' investigations if necessary. The data set was not anonymized in the reporting system but was anonymized before data analysis.

### 2.3 Statistical analysis

Medians and interquartile ranges (IQRs) were calculated for continuous variables and absolute numbers and proportions for categorical variables. Demographic, epidemiological and clinical characteristics were compared among four observed epidemic waves (March to April in 2013, December 2013 to May 2014, October 2014 to May 2015 and December 2015 to May 2016) and between surviving and dead patients infected with H7N9. According to the Diagnosis and Treatment Guideline for human infection with H7N9 published by National Health and Family Planning Commission of China, patients with no clinical symptoms and two consecutive PCR‐negative results (at least 24 hours of sampling interval) could be discharged from hospital; thus, the endpoint of disease duration for survived patients in our study was defined as the date of hospital discharge. The Pearson chi‐squared test with continuity correction was used for comparing proportions, or Fisher's exact test was used when appropriate. Mann‐Whitney *U* tests (for two groups) or Kruskal‐Wallis *H* tests (for multiple groups) were used for comparing medians. All study variables with statistical significance in univariate analyses between patients with survival and fatal outcome were included in multivariate analyses. A step‐forward logistic model was employed to select independent variables associated with fatal infection with H7N9 virus. Receiver‐operating characteristic (ROC) analysis was used to investigate the cut‐off value of disease duration for predicting cases' poor outcomes. Statistical analyses were done in r version 3.0.2 (R Foundation for Statistical Computing, Vienna, Austria) and statistical significance set at *P*≤.05. Information on missing data is shown in Table S1. Cases with missing data on variables being analysed were excluded.

## 3 RESULTS

**Figure 1:**
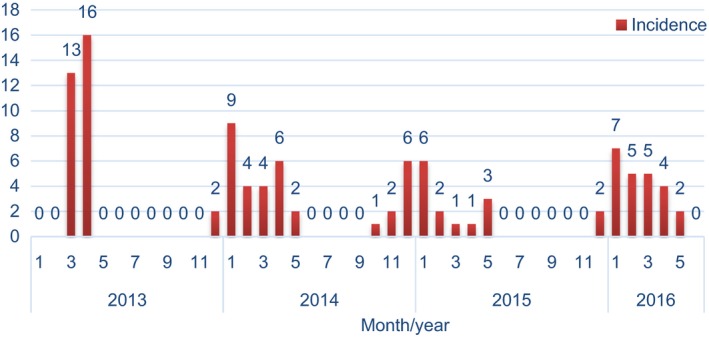
Four epidemic waves of human infections with H7N9 in Jiangsu Province, China, 2013‐2016

Four epidemic waves of human infections with H7N9 have been observed in Jiangsu Province, China: March to April in 2013 (wave 1), December 2013 to May 2014 (wave 2), October 2014 to May 2015 (wave 3) and December 2015 to May 2016 (wave 4) (Figure 1). The reported numbers of laboratory‐confirmed H7N9 cases were 29, 27, 22 and 25, respectively (Table 1). The median age of all cases was 54 years (IQR, 41‐68), and 69.9% were male. Epidemiological and clinical characteristics of human H7N9 cases were compared among the four waves. There were no statistically significant differences in age, gender or chronic medical conditions. Interestingly, both the proportion of overweight (from 10.7% to 40.0%, *P*=.029) and of rural residents (from 13.8% to 40.0%, *P*=.028) increased significantly across four epidemic waves. Significant differences were observed in time interval from onset of disease to laboratory confirmation, to onset of ARDS and of respiratory failure (*P*=.007, .017 and .004, respectively), but not in the initiation of or the time length of antivirals and glucocorticoids administration. The median number of days from onset of disease to administration of antivirals was 9 in the first wave and then decreased to 7 in the following three waves and was 7 days for both the survivors and fatal cases, with no statistically difference observed among the four waves, nor between survivors and fatal cases (*P*=.567, .980, respectively). The median days from onset of disease to laboratory confirmation decreased from 12 in wave 1 to 8 in waves 3 and 4. The median time intervals from onset of disease to onset of ARDS and of respiratory failure were both shortest during wave 3. Similarly, disease durations of fatal cases were also shortest during wave 3. No significant differences existed in poultry or live poultry market exposure history or in clinical complications among the four epidemic waves, except for heart failure. Half of the cases in wave 3 developed heart failure, while this proportion only ranged from 8.3% to 28.0% during the other epidemic waves (*P*=.023). A large majority of the cases were admitted to ICU (ranged from 74.1% to 85.0%) and treated with neuraminidase inhibitors (74.1%‐100%) and with antibiotics (95%‐100%). Notably, a significant increase in glucocorticoid administration was observed across four epidemic waves, from 61.5% to 100% (*P*=.010, *P* for trend =.001). In addition, the prescription of a higher dose of neuraminidase inhibitors (150 mg vs 75 mg) became more common (from 15.8% to 90.5%). The case fatality rate ranged from 34.5% to 61.9% across four epidemic waves, with no statistically significant difference (*P*=.287) (Table 1).

**Table 1:** Comparisons of selected demographic, epidemiological and clinical characteristics among four epidemic waves and between survival and dead patients infected with H7N9

Selected characteristics	Epidemic wave				*P* a	Clinical outcome		*P* b
	1 (N=29)	2 (N=27)	3 (N=22)	4 (N=25)		Survival (N=55)	Fatal (N=47)	
Demographic characteristics								
Male, n (%)	21 (72.4)	20 (74.1)	14 (63.6)	17 (68.0)	.858	40 (72.7)	31 (66.0)	.459
Age, y, median (interquartile)	54 (34‐71)	53 (42‐66)	57 (49.75‐69.25)	53 (38.5‐65)	.698	52.00 (36.00‐60.00)	60.00 (49.00‐73.00)	.**005**
Overweight, n (%)	3 (10.7)	9 (33.3)	6 (30.0)	10 (40.0)	.096	12 (21.8)	16 (35.6)	.128
Rural residence, n (%)	4 (13.8)	7 (25.9)	7 (31.8)	10 (40.0)	.175	16 (29.1)	12 (25.5)	.688
Chronic medical condition								
Chronic pulmonary disease, n (%)	5 (18.5)	1 (3.7)	4 (20.0)	2 (8.0)	.226^c^	3 (5.6)	9 (20.0)	**.028**
Chronic cardiovascular disease, n (%)	7 (25.9)	11 (40.7)	12 (60.0)	8 (32.0)	.102	14 (25.9)	24 (53.3)	**.005**
Chronic metabolic disease, n (%)	3 (11.1)	5 (18.5)	1 (5.0)	3 (12.0)	.573^c^	6 (11.1)	6 (13.3)	.736
Chronic liver disease, n (%)	1 (3.7)	2 (7.4)	2 (10.0)	2 (8.0)	.860^c^	4 (7.4)	3 (6.7)	1.000
Any medical condition, n (%)	14 (51.9)	17 (63.0)	13 (65.0)	13 (52.0)	.693	29 (53.7)	28 (62.2)	.393
Selected time intervals, d								
Time interval from onset of symptom to first medical consultation, median (interquartile)	2.00 (.00‐5.00)	3.00 (1.00‐4.00)	2.50 (.00‐5.75)	3.00 (.00‐4.50)	.911	2.50 (0.75‐5.00)	3.00 (0.00‐4.50)	.760
Time interval from onset of disease to hospital admission, median (interquartile)	5.00 (4.00‐8.50)	5.00 (4.00‐7.00)	5.00 (3.25‐7.75)	5.00 (3.50‐6.50)	.850	5.00 (4.00‐7.00)	5.00 (4.00‐7.00)	.759
Time interval from onset of disease to laboratory confirmation, median (interquartile)	12.00 (9.00‐15.00)	10.00 (8.00‐12.00)	8.00 (7.00‐12.00)	8.00 (7.00‐11.50)	.**007**	10.00 (8.00‐12.00)	10.00 (8.00‐13.00)	.895
Time interval from onset of disease to antivirals administration, median (interquartile)	9.00 (5.00‐14.00)	7.00 (6.00‐9.00)	7.00 (6.00‐9.00)	7.00 (5.00‐8.50)	.567	7.00 (5.00‐10.25)	7.00 (6.00‐9.00)	.980
Time interval from onset of disease to glucocorticoids administration, median (interquartile)	9.00 (5.00‐15.00)	8.00 (6.00‐10.00)	7.00 (5.25‐8.00)	7.00 (5.00‐8.00)	.258	7.00 (6.00‐11.50)	7.00 (5.00‐9.00)	.318
Time length of antivirals administration, median (interquartile)	12.00 (5.00‐15.00)	14.00 (6.00‐23.00)	9.50 (5.50‐13.75)	12.00 (7.50‐14.00)	.228	11.50 (7.00‐14.25)	13.00 (5.00‐19.50)	.598
Time length of glucocorticoids administration, median (interquartile)	7.00 (2.25‐15.25)	8.50 (4.25‐12.00)	8.50 (4.50‐18.00)	13.25 (5.00‐20.75)	.483	10.00 (3.50‐16.50)	7.00 (4.00‐18.00)	.685
Time interval from onset of disease to ICU admission, median (interquartile)	9.95 (6.46‐13.69)	9.07 (7.59‐10.87)	6.89 (5.60‐9.04)	7.62 (5.77‐10.06)	.104	8.92 (6.69‐11.46)	8.50 (6.04‐10.01)	.431
Time interval from onset of disease to onset of ARDS, median (interquartile)	8.50 (5.00‐14.00)	9.50 (8.00‐11.50)	6.00 (5.00‐8.00)	8.00 (6.00‐8.75)	**.017**	8.00 (6.00‐11.00)	8.00 (5.00‐10.00)	.260
Time interval from onset of disease to onset of Respiratory failure, median (interquartile)	8.00 (5.25‐14.00)	9.00 (7.50‐10.50)	6.00 (5.00‐8.00)	7.00 (5.00‐8.00)	**.004**	7.50 (6.00‐10.00)	8.00 (5.50‐10.00)	.810
Disease duration, d						31.00 (26.00‐46.00)	21.00 (17.00‐35.00)	<.**0001**
Survival, median (interquartile)	41.00 (26.00‐88.00)	29.50 (26.50‐44.00)	28.50 (21.00‐41.75)	29.00 (26.75‐39.50)	.318			
Dead, median (interquartile)	28.00 (20.00‐48.00)	24.00 (19.50‐41.00)	15.00 (12.50‐24.00)	21.00 (13.00‐27.00)	**.028**			
Poultry exposure								
Occupational exposure, n (%)	4 (14.8)	2 (7.4)	3 (15.0)	3 (13.6)	.908	9 (17.6)	3 (6.7)	.105
Direct contact with poultry, n (%)	6 (28.6)	14 (51.9)	8 (42.1)	13 (59.1)	.206	23 (50.0)	18 (41.9)	.441
Visit to live poultry market or poultry farm, n (%)	12 (60.0)	20 (74.1)	13 (68.4)	12 (54.5)	.504	29 (61.7)	28 (68.3)	.519
Any poultry exposure, n (%)	14 (51.9)	20 (74.1)	15 (75.0)	17 (77.3)	.168	34 (66.7)	32 (71.1)	.639
Clinical outcome								
ARDS, n (%)	16 (61.5)	18 (66.7)	16 (80.0)	16 (66.7)	.601	23 (44.2)	43 (95.6)	**<.0001**
Respiratory failure, n (%)	20 (76.9)	21 (80.8)	15 (78.9)	15 (65.2)	.603	30 (57.7)	41 (97.6)	**<.0001**
Liver dysfunction, n (%)	11 (45.8)	11 (42.3)	11 (55.0)	5 (20.8)	.116	15 (30.0)	23 (52.3)	**.028**
Renal dysfunction, n (%)	11 (44.0)	7 (26.9)	7 (35.0)	6 (25.0)	.468	9 (17.6)	22 (50.0)	**.001**
Heart failure, n (%)	7 (28.0)	7 (26.9)	10 (50.0)	2 (8.3)	**.023**	4 (7.8)	22 (50.0)	**<.0001**
Septic shock, n (%)	8 (33.3)	7 (26.9)	10 (50.0)	8 (33.3)	.427	6 (11.8)	27 (62.8)	**<.0001**
Fatality, n (%)	10 (34.5)	13 (48.1)	13 (61.9)	11 (44.0)	.287	NA		
Treatment								
Neuraminidase inhibitors, n (%)	20 (74.1)	27 (100.0)	20 (100.0)	21 (87.5)	**.006** c	47 (87.0)	41 (93.2)	.507
75 mg/per time, n (%)	16 (84.2)	9 (33.3)	5 (25.0)	2 (9.5)	**<.0001**	18 (39.1)	14 (34.1)	.630
150 mg/per time, n (%)	3 (15.8)	18 (66.7)	15 (75.0)	19 (90.5)		28 (60.9)	27 (65.9)	
Glucocorticoids, n (%)	16 (61.5)	20 (76.9)	16 (80.0)	24 (100.0)	**.010**	37 (69.8)	39 (90.7)	.**012**
Antibiotics, n (%)	25 (96.2)	27 (100.0)	19 (95.0)	25 (100.0)	.501^c^	51 (96.2)	45 (100.0)	.190^c^
ICU admission, n (%)	21 (77.8)	20 (74.1)	17 (85.0)	19 (79.2)	.842	35 (64.8)	42 (95.5)	<.**0001**

a Comparisons among four epidemic waves.

b Comparisons between survival and dead patients.

c Mann‐Whitney *U* tests (for two groups) or Kruskal‐Wallis *H* tests (for multiple groups) were used.

We further compared the epidemiological and clinical characteristics between all survival and fatal H7N9 cases. The median age of fatal cases was significantly greater than that of survivors (60 vs 52, *P*=.005). Chronic pulmonary disease (20.0% vs 5.6%, *P*=.028) and chronic cardiovascular disease (53.3% vs 25.9%, *P*=.005) were found more commonly in fatal cases than in survivors. No significant differences were observed in overweight, rural residence, chronic metabolic disease, chronic liver disease and poultry or live poultry market exposure history between fatal and surviving cases. There were no significant differences in selected time intervals between fatal and survival cases either, such as time interval from onset of symptom to first medical consultation, to laboratory confirmation or to onset of respiratory failure, the initial and time length of antivirals and glucocorticoids administration.

Disease duration was defined as the time interval from onset of disease to either hospital discharge or death. The median disease duration of fatal cases was considerably shorter than that of survivors (21 days vs 31 days, *P*<.0001). Fatal cases were more likely to be admitted into ICU and to have complications such as ARDS, respiratory failure, liver and renal dysfunction, heart failure and septic shock (*P*<.05). Administrations of antibiotics and neuraminidase inhibitors (including different doses) were not associated with fatal outcome, while glucocorticoids were administrated more frequently in fatal cases (*P*=.012).

All statistically significant factors found in univariate analyses aforementioned were included in a multivariate step‐forward logistic regression model to identify the independent predictors of fatal outcome of human infections with H7N9. Development of complications such as ARDS (OR=14.94, 95%CI: 1.82‐122.84), heart failure (OR=11.15, 95%CI: 0.95‐130.55) and septic shock (OR=22.97, 95%CI=1.79‐295.50) and administration of glucocorticoids (OR=34.11, 95%CI=1.62‐720.60) was found to be associated with an elevated risk of death, while prolonged disease duration was associated with a reduced risk (OR=0.91, 95%CI=0.85‐0.97). The combination of these factors could best predict death with an overall correct classification percentage of 94.4%. Furthermore, ROC analyses indicated that disease duration of 25.5 days could serve as the cut‐off value for predicting cases' poor outcomes (sensitivity=0.889, specificity=0.533).

## 4 DISCUSSION

H7N9 influenza virus continues to cause human infections and has resulted in four epidemic waves in Jiangsu Province, eastern China, up to June 2016. Both the proportion of overweight and of rural residents increased significantly across four waves in reported H7N9 patients. However, no significant difference in overweight between urban and rural H7N9 patients was observed (*P*=.937). Thus, the change in overweight might be attributed to the characteristics of subpopulations (cases were reported from different regions of Jiangsu Province across four waves) or to the overall rise of overweight rate in Jiangsu Province as a whole, rather than the involvement of more rural patients. According to the surveillance data of Jiangsu Provincial CDC, the overall obesity rate has increased from 9.5% to 14.6% from 2007 to 2013. It was reported that overweight could impair immunity against the influenza virus^9^ and was associated with an increased risk of severe infection.^10^ The increasing trend of overweight observed in the study has reminded public health experts of the importance of strengthening health education and of promoting precaution implementation in this high‐risk population. Several major cities afflicted by H7N9 in Jiangsu Province, such as Nanjing, Suzhou, Wuxi and Zhenjiang, suspended live poultry markets (LPMs) in urban areas to alleviate human infections.^11^ This may have boosted the live poultry trading in rural areas, leading to an increased number of rural H7N9 patients. A similar increasing trend was also observed in Zhejiang province, which was another hot spot of H7N9 epidemic in China.^12^

The time interval from onset of disease to laboratory confirmation of H7N9 patients has been shortened from a median of 12 days to 8 days across four epidemic waves. This might be attributed to improved laboratory capacity of municipal Centers for Disease Control and Prevention (CDC),^13^ avoiding repeated tests for confirmation by Provincial CDC.^3^ In addition, increased awareness of clinical doctors and the public might also be involved.^13^ A similar trend was also reported by Wu et al (2013) at the national level in China.^3^

It seemed that patients' disease progress was fastest during the third epidemic wave, as the time interval from onset of disease to onset of ARDS and to respiratory failure, and the disease duration of fatal cases were all shortest during this wave. The observed highest prevalence of chronic cardiovascular disease (60%) in reported H7N9 cases of this wave might be responsible. Incidence of heart failure was highest in cases of this wave as well (50%, *P*=.023). This indicates a critical role of cardiovascular function in the prognosis of human infections with H7N9.^14^, ^15^

A large majority of the cases were treated with neuraminidase inhibitors, and the implementation of a higher dose (150 mg) became the norm (from 15.8% to 90.5%). However, the higher dose was not found to be associated with a better prognosis in this study. Double dose of oseltamivir administration was originally recommended by WHO during the 2009 influenza A(H1N1) pandemic^16^ and was recommended for treating severe human infections with H7N9 in the Diagnosis and Treatment Guideline published by National Health and Family Planning Commission of China since 2013. As shown in our study, more and more clinicians have adopted this recommendation, but unfortunately no evidence of benefits was found. Yang et al. (2012)^17^ indicated that higher doses of oseltamivir did not improve clinical outcome in patients with pneumonia caused by influenza A pandemic (H1N1) virus. Clinical trials and prospective studies also found no virological or clinical advantages with double‐dose oseltamivir compared with standard dose in patients with severe influenza.^18^, ^19^ Our study implied a similar finding of this issue in the context of H7N9 infection. Moreover, reports of neuraminidase inhibitor resistance in the H7N9 virus call for a more optimized neuraminidase inhibitor use in clinical procedures.^20^, ^21^

We found in this study that clinical complications such as ARDS, heart failure and septic shock could pose an elevated risk of death from H7N9 infection. This finding is consistent with previous studies and case reports.^5^, ^6^, ^7^ A longer disease duration was found to be associated with a reduced risk of death in our study. Previous studies also reported that the disease duration of fatal cases was shorter than that of patients who recovered.^22^, ^23^ Infections with several avian influenza subtypes, such as H9N2, H7N7, H7N2, H7N3, H10N7 and H6N1, are commonly regarded as self‐limiting diseases,^24^ and essential supportive care has been proved to be effective for recovery.^25^, ^26^ For H7N9, studies showed that viral load peaked within the first 10 days after symptom onset during the acute exudative phase,^27^ and individuals' anti‐H7N9 antibodies could be detected from day 21, along with a significant decline of viral load.^28^ This indicated that infection with H7N9 can also be considered self‐limiting, which highlighted the critical role of supportive health care in treatment. We suggest that a disease duration of 25.5 days might serve as the time indicator for patients' survival.

Interestingly, administration of glucocorticoids was associated with a significantly elevated risk of death, while its clinical implementation jumped from a proportion of 61.5% to 100% across the observed four epidemic waves in this study. Glucocorticoid is mainly used as an anti‐inflammatory agent and is also an alternative for the treatment of septic shock.^29^, ^30^, ^31^ The administration of glucocorticoids thus reflects disease severity to some extent. However, as observed in this study, glucocorticoids have been used more and more comprehensively for treating H7N9 patient, not only restricted to these with special need. Treating influenza with glucocorticoids remains controversial. A number of studies have reported that use of glucocorticoids increased the death risk from H1N1^32^, ^33^, ^34^, ^35^ and from H5N1.^36^ Slower viral clearance of influenza A (H3N2) virus was observed in patients treated with systemic glucocorticoid.^37^ Furthermore, a systematic review and meta‐analysis using data from 19 studies of glucocorticoid treatment and human infection of influenza virus have concluded that glucocorticoids were related with mortality, nosocomial infection, longer mechanical ventilation and longer ICU stay.^38^ As a result, a much more rational use of glucocorticoids based on sturdy scientific evidences is urgently needed,^39^ for instance, assessing patients' disease progression with objective medical indicators along with the types, doses and administrative routes of glucocorticoids.

In conclusion, the differences in epidemiological and clinical characteristics across four observed epidemic waves of H7N9 in Jiangsu of China were reported in this study and predictors for fatal infection were investigated using multivariate analyses based on a relatively large sample size. These results help us better understand the evolution of H7N9 and help clinicians prejudge the prognosis of H7N9 patients. Suggestions for appropriate medication treatment such as neuraminidase inhibitors doses and glucocorticoids administration were also provided.

## CONFLICT OF INTEREST

None.
