# Supplementary material for: Predictors for fatal human infections with avian H7N9 influenza, evidence from four epidemic waves in Jiangsu Province, Eastern China, 2013‐2016
Source: Influenza Other Respir Viruses. 2017 Jul 26;11(5):418–24. doi: 10.1111/irv.12461 (PMC5596522; doi:10.1111/irv.12461)
Supplement: Supplementary file 1 [file IRV-11-418-s001.docx]

Supplementary Table S1. Missing data of selected characteristics

| Selected characteristics | Missing data (n) |
| --- | --- |
| **Demographic characteristics** |  |
| Male | 0 |
| Age | 0 |
| Overweight | 3 |
| Rural residence | 0 |
| **Chronic medical condition** |  |
| Chronic pulmonary disease | 4 |
| Chronic cardiovascular disease | 4 |
| Chronic metabolic disease | 4 |
| Chronic liver disease | 4 |
| Any medical condition | 4 |
| **Selected time intervals** days |  |
| Time interval from onset of symptom to first medical consultation | 4 |
| Time interval from onset of disease to hospital admission | 5 |
| Time interval from onset of disease to laboratory-confirmation | 0 |
| Time interval from onset of disease to antivirals administration | 6 |
| Time interval from onset of disease to glucocorticoids administration | 0 |
| Time length of antivirals administration | 0 |
| Time length of glucocorticoids administration | 0 |
| Time interval from onset of disease to ICU admission | 0 |
| Time interval from onset of disease to onset of ARDS | 0 |
| Time interval from onset of disease to onset of Respiratory failure | 0 |
| **Disease duration** | 1 |
| **Poultry exposure** |  |
| Occupational exposure | 7 |
| Direct contact with poultry | 14 |
| Visit to live poultry market or poultry farm | 15 |
| **Clinical outcome** |  |
| ARDS | 3 |
| Respiratory failure | 6 |
| Liver dysfunction | 6 |
| Renal dysfunction | 5 |
| Heart failure | 5 |
| Septic shock | 6 |
| Death | 1 |
| **Treatment** |  |
| Neuraminidases | 2 |
| Glucocorticoids | 4 |
| Antibiotics | 2 |
